# Early-onset diabetes involving three consecutive generations had different clinical features from age-matched type 2 diabetes without a family history in China

**DOI:** 10.1007/s12020-022-03144-2

**Published:** 2022-08-03

**Authors:** Da-Wei Wang, Jing Yuan, Fang-yuan Yang, Hai-Yan Qiu, Jing Lu, Jin-Kui Yang

**Affiliations:** 1 Department of Endocrinology, Beijing Tongren Hospital, Capital Medical University, Beijing, 100730 China; 2 Department of General Medicine, Beijing Tongren Hospital, Capital Medical University, Beijing, 100730 China; 3 Beijing Key Laboratory of Diabetes Research and Care, Beijing Diabetes Institute, Beijing, 100730 China

**Keywords:** Maturity-onset diabetes of the young, Early-onset diabetes, Three consecutive generations, Whole-exome sequencing

## Abstract

**Purpose:**

Early-onset, multigenerational diabetes is a heterogeneous disease, which is often simplistically classified as type 1 diabetes (T1D) or type 2 diabetes(T2D). However, its clinical and genetic characteristics have not been clearly elucidated. The aim of our study is to investigate the clinical features of early-onset diabetes involving three consecutive generations (eDia3) in a Chinese diabetes cohort.

**Methods:**

Of 6470 type 2 diabetic patients, 105 were identified as eDia3 (1.6%). After a case–control match on age, we compared the clinical characteristics of 89 eDia3 patients with 89 early-onset T2D patients without a family history of diabetes (eDia0). WES was carried out in 89 patients with eDia3. We primarily focused on 14 known maturity-onset diabetes of the young (MODY) genes. Variants were predicted by ten tools (SIFT, PolyPhen2_HDIV, PolyPhen2_HVAR, LRT, Mutation Assessor, Mutation Taster, FATHMM, GERP++, PhyloP, and PhastCons). All suspected variants were then validated by Sanger sequencing and further investigated in the proband families.

**Results:**

Compared to age-matched eDia0, eDia3 patients had a younger age at diagnosis (26.5 ± 5.8 vs. 29.4 ± 5.3 years, *P* = 0.001), lower body mass index (25.5 ± 3.9 vs. 27.4 ± 4.6 kg/m^2^, *P* = 0.003), lower systolic blood pressure (120 ± 15 vs. 128 ± 18 mmHg, *P* = 0.003), and better metabolic profiles (including glucose and lipids). Of the 89 eDia3 patients, 10 (11.2%) carried likely pathogenic variants in genes (*KLF11*, *GCK*, *ABCC8*, *PAX4*, *BLK* and *HNF1A*) of MODY.

**Conclusions:**

eDia3 patients had unique clinical features. Known MODY genes were not common causes in these patients.

## Introduction

A remarkable increase in the prevalence of early-onset diabetes has become a new global trend [1], especially in Asia [2]. According to a national cross-sectional study, the prevalence of diabetes among adults younger than 40 years old was 5.7% in China [2]. Early-onset diabetes is a complicated, heterogeneous disease that is not simply divided into type 1 diabetes (T1D) or type 2 diabetes (T2D). Actually, the range of diabetes subgroups is becoming even more diverse, especially for early-onset, multigenerational diabetes, which has a considerable genetic predisposition. In order to obtain a precise diagnosis and better treatment strategy, deeper investigation of the clinical features and genetic backgrounds for early-onset diabetes involving three consecutive generations (eDia3) is critical for clinic practice.

As the most common type of monogenic diabetes in early-onset, multigenerational diabetes patients, maturity-onset diabetes of the young (MODY) was first reported in 1974 by Tattersall as mild familial diabetes with dominant inheritance [3]. Molecular genetic diagnosis of MODY has been recognized since the 1990s, and the mutations of the disease were identified after that. Previous studies suggested that MODY probably accounts for 1–5% of overall diagnosed diabetes [4, 5], with the most commonly reported subtypes as *GCK-*MODY (MODY2), *HNF1A*-MODY (MODY3), and *HNF4A*-MODY (MODY1).

There were clinical criteria to screen diabetic patients for genetic diagnosis of MODY. Previous classic guidelines identify candidates for performing MODY genetic testing including age at diagnosis typically before 25 years, non-insulin-dependent, and family history of diabetes of at least two generations [6]. The latest American Diabetes Association (ADA) guideline recommended MODY should be screened and further confirmed in diabetic patients with the following conditions: 1) diabetes without typical features of T1D or T2D; 2) stable, mild fasting hyperglycemia of 5.5–8.5 mmol/L) or stable glycated hemoglobin A1c (HbA1c) of 5.6–7.6% [7]. However, not all patients with MODY fulfill these criteria. To date, there are no worldwide sufficient and accepted criteria for selecting patients to undergo genetic testing. Although clinicians and researchers have recognized the significance of MODY, only a small number of studies have been conducted in China to select MODY through a large diabetes cohort based on a strict screening flowchart, and the prevalence and the genetic spectrum of MODY were still not fully elucidated.

To our knowledge, there has been no study comparing the clinical features of eDia3 with age-matched early-onset T2D patients without a family history of diabetes (eDia0), which may be due to different pathogenic backgrounds. Therefore, in a large hospital-based diabetes cohort from China, we aimed to investigate the clinical characteristics of eDia3, and in addition, to evaluate the genetic spectrum by whole exome sequencing.

## Research design and methods

### Participants and clinical characterization

**Fig. 1:**
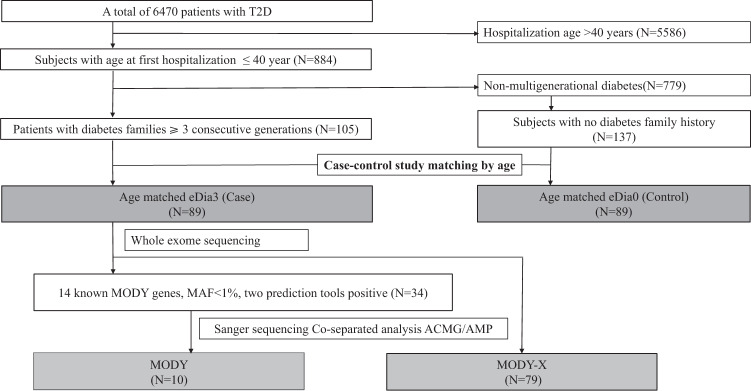
Flow chart of the study. T2D, type 2 diabetes; eDia3, early-onset diabetes involving three consecutive generations; eDia0, early-onset type 2 diabetes without a family history of diabetes; MODY, maturity-onset diabetes of the young

This study was performed in Han Nationality of Chinese Population. Among a hospital-based cohort of 6470 patients with T2D (according to ADA 2003 criteria) from January 2013 to December 2018 in Beijing Tongren Hospital, Capital Medical University (Beijing, China), 884 young early-onset patients with age at first hospitalization ≤40 years were enrolled in the study. Patients with secondary diabetes mellitus, gestational diabetes mellitus, type 1 diabetes or type 1 diabetes antibody positive, or other severe systemic diseases were excluded. Of the 884 young patients, 137 patients without a family history of diabetes and 105 probands with diabetes in three consecutive generations from unrelated families were further selected. Finally, with a case–control approach matching age at first hospitalization, 89 patients with eDia3 (Case) and 89 patients with eDia0 (Control) were included and compared. The flowchart of the study was shown in Fig. 1.

Clinical information was obtained for these young diabetes patients, including demographic information, diabetes history, and physical examinations (measurements of height, weight, systolic blood pressure (SBP), diastolic blood pressure (DBP), and waist and hip circumference) at the time of first hospitalization. Body mass index (BMI) and waist–hip ratio (WHR) were calculated. Laboratory tests included fasting plasma glucose, blood urea nitrogen, creatinine, uric acid, total bilirubin, direct bilirubin (DBIL), total cholesterol, low-density lipoprotein cholesterol, high-density lipoprotein cholesterol (HDL-C), triglycerides (TG), and high-sensitivity C-reactive protein for all participants, were measured by an automated biochemical analyzer (Beckman Coulter, Carlsbad, CA, USA). HbA1c was measured by high-performance liquid chromatography (VARIANT, Bio-Rad Lab. Hercules, CA, USA). C-peptide was measured by the method of electrochemiluminescence (Cobas e601; Roche Diagnostics, Tokyo, Japan). Homeostatic model assessment indices for beta-cell function or for insulin resistance were computed with fasting glucose and C-peptide levels using the HOMA2 calculator [8].

This study was approved by the Ethics Committee of Beijing Tongren Hospital, Capital Medical University (approval # TRECKY2009-36.), and was performed according to the principles of the Declaration of Helsinki II. Informed consent was obtained from each participant or the next of kin.

### Whole-exome sequencing (WES)

WES was carried out in patients with eDia3. Genomic DNA was extracted from peripheral blood using the TIANamp Blood DNA Maxi Kit for Mammalian Blood (Tiangen Biotech Co., Beijing, China). The DNA concentration was detected by Qubit Fluorometer. The optical density value ranged from 1.8 to 2.0, the DNA concentration was more than 12.5 ng/μl, and the DNA samples with a content of more than 1 μg could be used to build a library. The qualified genomic DNA sample was randomly fragmented by Covaris technology and the size of the library fragments was mainly distributed between 150 bp and 250 bp. Agilent V6 was used to hybridize and capture the DNA fragments of exon region, and the library was established. The library was sequenced using BGISEQ-500 sequencing platforms. The clean data were produced by data filtering on raw data. All clean data of each sample were mapped to the human reference genome GRGh37 (hg19). Burrows-Wheeler Aligner [9, 10] software was used to do the alignment. To ensure accurate variant calling, we followed recommended Best Practices for variant analysis with the Genome Analysis Toolkit (GATK, https://gatk.broadinstitute.org/hc/en-us). Local realignment around InDels and base quality score recalibration were performed using GATK (v3.7) [11, 12], with duplicate reads removed by Picard tools (http://broadinstitute.github.io/picard/). The depth and coverage for each individual were calculated based on the alignments. The effective sequencing depth of WES was ≥100 × depth.

### Read mapping, variant annotation, filtering, and classification

Among the genes covered by WES, we primarily focused on 14 known MODY genes, including *HNF4A*, *GCK*, *HNF1A*, *PDX1*, *HNF1B*, *NEUROD1*, *KLF11*, *CEL*, *PAX4*, *INS*, *BLK*, *ABCC8*, *KCNJ11*, and *APPL1* [13–21]. All genomic variations, including SNPs and InDels were detected by Haplotype Caller of GATK (v3.7) [11, 12]. Subsequently, the hard-filtering method was applied to get high-confident variant calls. The SnpEff tool (http://snpeff.sourcet/SnpEff_manual.html) and VEP tool (https://asia.ensembl.org/Tools/VEP) were applied to perform a series of annotations for variants. (1) Gene-based annotation: identify whether SNPs or InDels cause protein-coding changes and the amino acids that are affected. (2) Filter-based annotation: identify variants that are reported in dbSNP v151 (http://www.ncbi.nlm.nih.gov/snp/), and identify the subset of variants with minor allele frequency (MAF) < 1% in the 1000 Genome Project (http://www.1000genomes.org/) or gnomAD (http://gnomad.broadinstitute.org). These variants were further filtered to include those predicted to be damaging by ten prediction tools, including seven functional prediction tools (SIFT, PolyPhen2_HDIV, PolyPhen2_HVAR, LRT, Mutation Assessor, Mutation Taster, and FATHMM) and three conservation tools (GERP++, PhyloP, and PhastCons). The positive results of these prediction tools are defined as follows: SIFT < 0.05 (http://sift.bii.atar.edu.sg/) [22], PolyPhen2_HDIV > 0.453, PolyPhen2_HVAR > 0.447 [23] (http://genetics.bwh.harvard.edu/pph2), LRT = D (deleterious) [24], Mutation Assessor >1.938 [25], Mutation Taster = A (disease-causing automatic) or D (disease causing) [26], FATHMM < −1.5 [27], GERP++ > 3 [28], PhyloP > 2.5 [29], PhastCons > 0.6 [30]. Ten tools were used to ensure the comprehensiveness of the results. At least one of the seven functional prediction tools and one of the three conservation tools were positive, we reserved this variant. However, the results were just as a reference, not a judgment. All remaining variants were then validated by Sanger sequencing and further investigated in the proband families.

### Sanger sequencing

The remaining variants identified from the WES analysis were further validated using Sanger sequencing. Genomic DNA was extracted from probands with suspected variants and their family members using a TIANamp Blood DNA Midi Kit (Tiangen Biotech Co., Beijing, China) according to the manufacturer’s instructions. The variants were amplified from genomic DNA by polymerase chain reaction (PCR) using gene-specific primers. The PCR amplification was performed using a TechNet Genius Thermo Cycler (TechNet Inc., Princeton, NJ, USA) and the following cycling program: initial denaturation at 95 °C for 10 min; 35 cycles of denaturation at 95 °C for 30 s, annealing for 30 s (annealing temperatures are listed in File S1), and extension at 72 °C for 30 s; and a final extension at 72 °C for 5 min. The resulting PCR products were sequenced using an ABI3730XL instrument (Applied Biosystems) and the DNA sequences were compared using the Sequencer software (Gene Codes Corp., Ann Arbor, MI, USA).

### Statistical analysis

All statistical analyses were performed with SPSS ver. 17.0 software (SPSS Inc., Chicago, IL, USA). Student’s *t* test and the Wilcoxon rank test were used to compare continuous variables. For categorical variables, the chi-square test was used to analyze differences between two groups. *P* < 0.05 was considered statistically significant.

## Results

### Characteristics of the diabetic participants

The average age of all 6470 diabetic patients was 57.7 ± 13.0 years. According to our selection criteria, 13.7% (884/6,470) of patients were early-onset diabetes whose first hospitalization age was ≤40 years, and the average age of diabetes diagnosis was 31.2 ± 6.4 years. A total of 11.9% (105/884) of early-onset diabetic patients were eDia3, while 15.5% (137/884) were eDia0. The flowchart of the study is shown in Fig. 1.

### Clinical features of patients with eDia3

As shown in Supplemental Table 1, compared to early-onset diabetes patients without family history, early-onset diabetes patients with three generations of family history had a younger diagnosed age, longer duration, lower BMI, WHR, SBP, TG, DBIL, and HbA1c, but higher HDL and FBG. In age-matched early-onset diabetic patients, similar clinical features were found, those with eDia3 had a younger age at diagnosis (26.5 ± 5.8 vs. 29.4 ± 5.3 years, *P* = 0.001), lower BMI (25.5 ± 3.9 vs. 27.4 ± 4.6 kg/m^2^, *P* = 0.003), WHR (0.88 ± 0.11 vs. 0.94 ± 0.05, *P* < 0.001), and SBP (120.49 ± 15.29 vs. 128.04 ± 17.76 mmHg, *P* = 0.003), and better metabolic profiles (including lower TG (1.40 (1.04–2.48) vs. 1.82 (1.24–3.49) mmol/l, *P* = 0.023) and HbA1c (8.67 ± 2.14 vs. 10.06 ± 2.19%, *P* < 0.001), but higher HDL-C (1.09 ± 0.32 vs. 0.92 ± 0.29 mmol/l, *P* < 0.001) than patients with eDia0 (Table 1).

**Table 1:** Comparison of clinical characteristics of early-onset diabetic patients involving three consecutive generations (eDia3) or without a family history of diabetes (eDia3)

	Age matched cases and controls			eDia03		
	Control (eDia0) (*n* = 89)	Case (eDia03) (*n* = 89)	*P*	MODY (*n* = 10)	Non-MODY (*n* = 79)	*P*
Age at admission (yrs)	31.9 ± 5.3	31.5 ± 6.0	0.600	29.0 ± 8.76	31.7 ± 5.62	0.180
**Age at diagnosis (yrs)**	**29.4** ± **5.3**	**26.5** ± **5.8**	**0.001**	25.9 ± 7.8	26.6 ± 5.5	0.733
**Duration of diabetes (yrs)**	**1.0 (0.3–4.0)**	**4.0 (1.0–8.0)**	**0.000**	2.0 (0.8–4.2)	4.0 (1.0–9.0)	0.379
Male (%)	56 (62.9%)	49 (55.1%)	0.286.	6 (60.0%)	43 (54.4%)	0.739
**BMI (kg/m**^**2**^**)**	**27.4** ± **4.6**	**25.5** ± **3.9**	**0.003**	**23.2** ± **3.1**	**25.8** ± **3.9**	**0.046**
**WHR**	**0.94** ± **0.05**	**0.88** ± **0.11**	**0.000**	0.87 ± 0.06	0.88 ± 0.11	0.793
**HbA1c (%)**	**10.1** ± **2.2**	**8.7** ± **2.1**	**0.000**	9.5 ± 2.8	8.5 ± 2.04	0.182
FPG (mmol/l)	8.7 ± 3.2	9.7 ± 4.0	0.065	9.6 ± 3.1	9.7 ± 4.1	0.943
**SBP (mmHg)**	**128.0** ± **17.8**	**120.5** ± **15.3**	**0.003**	121.1 ± 13.6	120.4 ± 15.6	0.894
**DBP (mmHg)**	**81.1** ± **12.7**	**77.5** ± **9.3**	**0.031**	78.9 ± 11.4	77.3 ± 9.1	0.609
C-peptide (ng/ml)	2.1 ± 1.1	2.1 ± 0.8	0.760	1.8 ± 0.5	2.1 ± 0.9	0.411
CR (umol/l)	65.6 ± 17.8	65.5 ± 21.9	0.992	64.3 ± 17.5	65.7 ± 22.5	0.852
**BUN (mmol/l)**	**4.2** ± **1.5**	**4.9** ± **1.7**	**0.015**	4.4 ± 1.4	4.9 ± 1.8	0.401
**UA (mmol/l)**	**373** ± **94**	**343** ± **78**	**0.020**	331 ± 61	345 ± 80	0.616
TBIL (umol/l)	15.3 ± 5.6	14.7 ± 6.1	0.528	13.1 ± 3.5	14.9 ± 6.3	0.382
DBIL (umol/l)	2.6 ± 1.1	2.3 ± 1.2	0.078	2.1 ± 0.8	2.3 ± 1.3	0.611
hsCRP (ng/ml)	1.4 (0.5–3.7)	2.0 (0.8–5.6)	0.302	2.2 (0.4–6.6)	1.9 (0.8–5.4)	0.967
TC (mmol/l)	5.18 ± 1.55	4.86 ± 1.23	0.134	4.61 ± 1.02	4.89 ± 1.26	0.505
**TG (mmol/l)**	**1.8 (1.2–3.5)**	**1.4 (1.0–2.5)**	**0.023**	1.2 (1.0–1.8)	1.4 (1.1–2.5)	0.409
LDL (mmol/l)	3.11 ± 1.10	3.00 ± 1.00	0.461	3.18 ± 0.89	2.97 ± 1.02	0.539
**HDL (mmol/l)**	**0.92** ± **0.29**	**1.09** ± **0.32**	**0.000**	1.04 ± 0.16	1.10 ± 0.34	0.540
HOMA-β	65.7 ± 52.9	53.6 ± 39.1	0.087	48.0 ± 36.0	54.4 ± 39.7	0.631
HOMA-IR	1.87 ± 1.00	1.95 ± 1.07	0.614	1.66 ± 0.44	1.99 ± 1.12	0.372
Treatment, n (%)						
OADs	41 (46.07%)	36 (40.44%)	0.449	7 (70%)	33 (41.77%)	0.475
Insulin/OADs+Insulin	48 (53.93%)	53 (59.55%)		3 (30%)	46 (58.22%)	

*BMI* body mass index, *WHR* waist hip ratio, *HbA1c* glycated hemoglobin, *FPG* fasting plasma glucose, *SBP* systolic blood pressure, *DBP* diastolic blood pressure, *CR* creatinine, *BUN* blood urea nitrogen, *UA* uric acid, *TBIL* total bilirubin, *DBIL* direct bilirubin, *hsCRP* high sensitivity C-reactive protein, *TC* total cholesterol, *TG* triglycerides, *LDL* low-density lipoprotein cholesterol, *HDL* high-density lipoprotein cholesterol, *OADs* oral antidiabetic drugs, *HOMA-β* homeostatic model assessment indices for beta-cell function, *HOMA-IR* homeostatic model assessment indices for insulin resistance

The significance of the bold values represent the data are statistically significance with a *P* value < 0.05

### Variant classification and prevalence of clinically suspected MODY

A total of 21 rare , nonsilent variants (at least two of ten prediction tools were positive , MAF < 1%) were identified in 8 MODY‑related genes (from 34 probands) (Supplementary Table 1). After assessing the likelihood of causality by using the ACMG/AMP guidelines and investigated in the proband families, of the 21 rare, nonsilent variants, 6 variants were identified as likely benign, 5 variants were classified as having uncertain significance, and 10 variants remained as likely pathogenic with MAF < 0.0001. Of the ten variants classified as pathogenic/likely pathogenic, eight were novel, and two have been previously reported to cause MODY (Table 2).

**Table 2:** List of variants identified pathogenic/likely pathogenic

Patient	Age	sex	BMI	Gene	Transcript	Codon	Variant	dbSNP	Frequency	SIFT	PolyPhen2	Significance	Ref.
P-1	27	M	22	*KLF11*	NM_003597.4:p.Gly172Arg/c.514G>A	Ggg/Agg	missense	rs1351414401	0.000012	D	B	likely-pathogenic	—
P-2	20	M	25	*KLF11*	NM_003597.4:p.Glu265Lys/c.793G>A	Gaa/Aaa	missense	rs773720978	0.00004	D	B	likely-pathogenic	—
P-3	32	F	25	*KLF11*	NM_003597.4:p.Gly251Glu/c.752G>A	gGg/gAg	missense	rs758135671	0.00001	T	PD	likely-pathogenic	—
P-4	28	M	25	*PAX4*	NM_006193.2:p.Arg12Trp/c.34C>T	Cgg/Tgg	missense	rs149708455	0.00008243	D	D	likely-pathogenic	—
P-5	33	M	19	*BLK*	NM_001715.2:p.Met121Ile/c.363G>A	atG/atA	missense	rs1334858946	0.000004	D	B	likely-pathogenic	—
P-6	11	F	23	*ABCC8*	NM_001287174.1:p.Val784Met/c.2350G>A	Gtg/Atg	missense	rs764239078	0.00000824	D	PD	likely-pathogenic	—
P-7	36	M	28	*ABCC8*	NM_001287174.1:p.Lys134Thr/c.401A>C	aAg/aCg	missense	rs762524562	0.000008	D	D	likely-pathogenic	—
P-27	30	F	24	*GCK*	NM_000162.3:p.Asn392Lys/:c.1173C>A	Ctt/Att	missense	rs1554334579	—	D	D	likely-pathogenic	—
P-28	17	F	17	*GCK*	NM_000162.3:p.Leu77Arg/c.230T>G	cTg/cGg	missense	—	—	D	D	likely-pathogenic	[53]
P-31	25	M	25	*HNF1A*	NM_000545.6:p.Arg203His/c.608G>A	cGt/cAt	missense	rs587780357	0.000008	D	D	likely-pathogenic	[54]

SIFT: D, Deleterious (< .05); T, Tolerated (>=0.05)

PolyPhen2: D, Damaging (>=0.957); PD, Possibly Damaging (0.453 < =pp2 < =0.956); B, Benign (<=0.452)

**Fig. 2:**
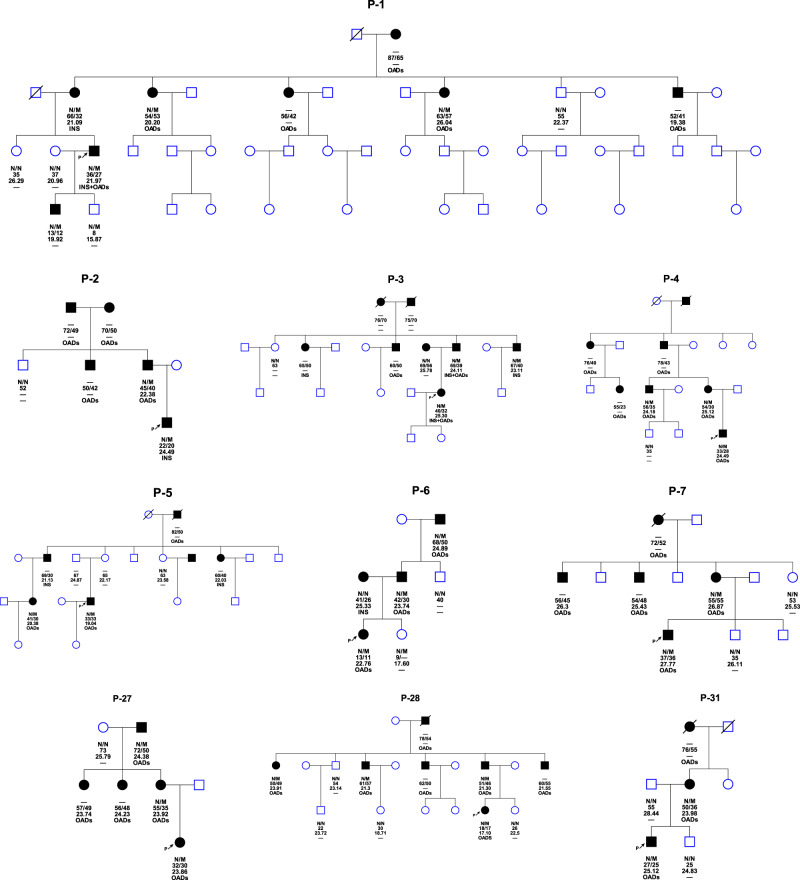
Family hierarchical diagram of the genetic confirmed MODY. Squares denote male family members and circles denote female family members. Solid symbols represent subjects with diabetes and open symbols represent nondiabetic individuals. The genotype is shown underneath each symbol. N/M denotes mutation, while N/N denotes no mutation. Below the genotype are age in years at observation, age in years at diabetes diagnosis, then the BMI and the specific anti-hyperglycemic treatment. Arrow indicates the proband of the family. Ins, insulin treatment; OADs, oral anti-diabetes drugs

Overall, likely pathogenic MODY‑related genetic variants were identified in 11.2% (10/89) of eDia3 patients and in 1.13% (10/884) of all early-onset diabetes patients. The Sanger sequencing results for the ten probands with suspected variants and their family members are shown in Fig. 2. In these families, suspected variants were confirmed in probands and cosegregation with disease in multiple affected family members. Out of ten likely pathogenic MODY‑related genetic variants, three novel *KLF11* mutations were found in three probands. Variants of the *KLF11* gene (3 cases) were the most common subtype of MODY in this study, followed by variants of *GCK* (2 cases), *ABCC8* (2 cases), *PAX4* (1 case), *BLK* (1 case), and *HNF1A* (1 case)

## Discussion

Early-onset diabetes is a highly heterogeneous group of disorders, differential diagnosis of early-onset type 2 diabetes remains difficult [31]. As we know, the development of early onset T2D represents a complex interplay between genetic and environmental factors. Obesity, low physical activity, high sedentary behavior, socioeconomic status, ethnicity, family history, low birth weight, exposure to diabetes mellitus in the uterus are all factors for developing early onset T2D [32]. In addition, previous studies had reported that aberrant fetal programing seemed to increase the risk of diabetes [33]. Nevertheless, compared with eDia0 who have less genetic background and are mainly affected by environmental factors, eDia3 patients are affected by both genetic and environmental factors. Proper classification of these patients is a major challenge to clinicians. A previous study defined the multigenerational form of diabetes mellitus as “familial diabetes of adulthood” (FDA) [34] and revealed significant clinical differences between FDA and T2D. In this study, we performed an extreme case–control study with patients with eDia3 as cases and those with eDia0 as controls. Although the age of diabetes diagnosis was under 40 years, statistically significant differences in diabetes onset age, duration, BMI, and metabolic biomarkers were found between the two groups. The results suggested a hypothesis of different pathogenetic backgrounds between the two subgroups.

The reason for the longer disease duration in eDia3 at the first admission may be related to the delay of the first hospitalization which due to the milder clinical and metabolic indicators in the early stage of the disease in eDia3. Previous studies have found that monogenic diabetes was more prevalent in the milder phenotypic cluster. Our study revealed that eDia3 had better metabolic phenotype than age-matched eDia0, even with younger diagnosed age and longer duration of diabetes. The mechanism underlying this phenomenon was still unclear. Lower BMI and WHR of eDia3 may be an explanation. As we know, obesity, especially abdominal obesity, usually is more prone to metabolic abnormalities such as increased blood pressure and abnormal blood lipids. Besides, other unknown factors may also lead to current results, which need comprehensive studies in the future.

In the past, the most common screening process used to identify candidates for performing MODY genetic testing, included age at diagnosis typically before 25 years, noninsulin dependence, and family history of diabetes with at least two generations [6]. However, a study selected 1564 probands and reported that using stringent inclusion criteria would miss 70% of cases of monogenic diabetes [31]. Meanwhile, age, obesity, insulin resistance, and other nongenetic factors can modify clinical presentation of MODY, remarkable overlaps of characteristics were observed between MODY and T1D/T2D patients. Therefore, this study detected MODY in patients with eDia3 and did not restrict the weight of the patients. Potential clinical biomarkers were investigated to help prioritize the strategy of selecting diabetes patients for genetic testing [35]. Although our study found that eDia3 is significantly different from eDia0 in clinical characteristics, it was also found that these clinical indicators could not be used as a precise biomarker for known MODY screening.

WES was carried out in 89 patients with eDia3 and the findings demonstrated that variants of genes related to MODY1-14 were not mainly causing for patients with eDia3 in China. The genetic confirmed MODY was detected in 11.2% patients with eDia3 (10/89) and only in 1.13% early-onset diabetes patients (10/884). These results are comparable to a Korean study which found a prevalence of 12.8% in the four relatively common MODY genes (*HNF1A*, *HNF4A*, *HNF1B*, or *GCK*) among 109 diabetes patients with onset age ≤30 years and a BMI ≤ 30 kg/m^2^ [36]. Similarly, an UK study demonstrated that the mutation pick-up rate of MODY genes (*HNF1A*, *HNF4A*, *HNF1B*, or *GCK*) in South Asian participants was 12.6%, lower than White European group (25.2%) [37]. Conversely, a recent Chinese study selected 42 clinically diagnosed MODY aged ≤18 years and identified 24 patients (57.1%) had mutations in the known MODY genes [38]. The discrepancy in the MODY detection rate may partially contributed to the ethnic differences [39] and the clinical criteria used to select participants for genetic testing, leading to a variety of baseline characteristics varies a lot in different studies. To date, most studies have searched for genetic causes of MODY in Euro Caucasian patients, while only a small number of studies have been conducted in Arabia and the Middle East. [40, 41] In recent years, although the relevant studies carried out in Asian populations have gradually increased, but more common in Japan and South Korea [36, 42, 43], and there are fewer large sample studies in Han Nationality of Chinese Population [44, 45]. This study demonstrated that mutations of genes related to MODY1-14 were not the main cause of eDia3 in Chinese patients, which indicated that the pathogenic background of eDia3 needs further investigation in the future.

Except the 10 variants that are likely pathogenic to MODY, we also found 11 rare, non-silent variants in 24 patients, classified as likely-benign or uncertain significance. Of which, the variants of *PAX4* were identified in 16 patients, with *PAX4* Arg192His variant (chr7:127253550, rs2233580) in 8, *PAX4* Arg192Ser variant (chr7:127253551, rs3824004) in 5, and *PAX4* Arg31Gln variant (chr7:127255483, rs115887120) in 3. *PAX4* is a transcription factor that plays an crucial role in beta cell development, differentiation, and survival [46]. It had been suggested that the mutations of *PAX4* gene were positively and ethnic-specifically associated with the risk of T2D in Asian population [47]. Genome-wide association studies in Chinese populations identified *PAX4* arg192his (rs2233580) as a T2DM susceptibility locus [48]. A Korean study found that the combination of *PAX4* Arg192His and *PAX4* Arg192Ser could be considered a strong risk factor for T2D, and having two copies of *PAX4* Arg192His variant was related to a 7.0 years earlier onset of diabetes [49]. Other studies also provided evidence that missense variant rs2233580 (p.Arg192His) in *PAX4* gene was significantly associated with T2D, which is related to the reduction of C-peptide and the age of diagnosis in T2D patients [50] Combined with the occurrence of *PAX4* arg192his (rs2233580) genotype in eDia3 patients in this study, it is also confirmed that it may be a high-risk genetic factor for eDia3 in China [48].

Previous study suggested *HNF1A*-MODY (53%), *GCK*-MODY (32%) were most common subtypes of MODY [51]. However, the etiology of the MODY in our study demonstrated that variants of *KLF11* genes were more frequently involved. Chinese research also identified the prevalence of *HNF1A*-MODY and *GCK*-MODY was only 9% and 1% in patients with suspected MODY [52]. The prevalence of rare subtypes of MODY was relatively high in patients with eDia3 in our study. The cause of the variation in the frequencies of mutations between our data and previous reports remains unclear. The different genetic background might be an important reason for the phenomena. Our findings indicated that the pathogenic background of hyperglycemia had not been elucidated in vast majority of patients with eDia3, especially expanding age and BMI standards, which require further and broader attempts and get deeper insight into the molecular causes in the future investigation.

Mutations in *KLF11* may lead to the development of MODY7, which appeared to be involved in impaired insulin secretion. It was first reported in early-onset T2D patients with two rare variants (Ala347Ser and Thr220Met) [17]. To date, only a few studies identified mutations of *KLF11* gene in screening MODY. In 2019, a Japanese study reported a novel *KLF11* variant (p.His418Gln) which was linked to early childhood-onset type 1B diabetes. (Ushijima K, Narumi S, Ogata T, Yokota I, Sugihara S, Kaname T, Horikawa Y, Matsubara Y, Fukami M, Kawamura T; Japanese Study Group of Insulin Therapy for Childhood and Adolescent Diabetes. KLF11 variant in a family clinically diagnosed with early childhood-onset type 1B diabetes. Pediatr Diabetes. 2019 Sep;20(6):712–719.) Moreover, previous studies demonstrated mutations of *KLF11* p.Lys453del [50], *KLF11* (p.I89L and p.G484S) [51], and *KLF11* (c.1061G>T) (Clinical and Functional Characteristics of a Novel KLF11 Cys354Phe Variant Involved in Maturity-Onset Diabetes of the Young) in Chinese population. *KLF11*-MODY is extremely rare and seemed to be more prevalent in Asian population. Up to now, there is no large-scale researches and summary of clinical characteristics of MODY7. The probands from the above studies exhibited hyperglycemia at ages from 1 to 23 years, and observed to be negative for islet cell autoantibodies. Findings from our study demonstrated that the mutations in *KLF11* gene were not rare form of MODY in this Chinese cohort, with three novel heterozygous missense mutations (Gly172Arg for P-1; Glu265Lys for P-2; Gly251Glu for P-3). The probands had an average diagnosed age of 26.3 years and BMI of 24 kg/m^2^. The results of our study suggested that the clinical phenotype is less well defined and it was of critical significance to screen rare subtypes of MODY in Chinese subjects.

There are some limitations of our study. First, it was a hospital-based study including patients with relatively high HbA1c and increased prevalence of diabetic vascular complications. Therefore, patients with mild asymptomatic hyperglycemia could not be selected in our study, which may influence the detection rate of gene mutations. Second, WES test of the eDia0 cohort was not carried out in this study according to the guidelines [6] and medical ethics, it is, therefore, unclear whether there were genetically diagnosed MODY patients in the control group. Third, some relatives of the genetic confirmed MODY patients could not be connected to perform the genetic testing. In some cases, due to the unavailable information of all family members related to the probands, we could not perform a segregation analysis of some rare potentially pathogenic variants identified in our study. Fourth, this study only included Chinese participants and ethnic differences might exert an important effect on the diagnosis rate and genotype of MODY.

In summary, eDia3 patients had different clinical characteristics from age-matched T2D patients. Known MODY genes were not common causes of clinically suspected MODY, and *KLF11* gene mutations were more frequently identified in these patients in China. The reasons for these findings cannot be fully explained by our current study. Hence, more comprehensive studies are needed.

## Supplementary information

Supplemental Table 1-R1
