# Supplementary material for: Early-onset diabetes involving three consecutive generations had different clinical features from age-matched type 2 diabetes without a family history in China
Source: Endocrine. 2022 Aug 3;78(1):47–56. doi: 10.1007/s12020-022-03144-2 (PMC9474578; doi:10.1007/s12020-022-03144-2)
Supplement: Supplementary file 1 — Supplemental Table 1-R1 [file 12020_2022_3144_MOESM1_ESM.docx]

**Supplemental Table 1.** Demographic and clinical characteristics of early-onset diabetes patients with three generations of family history and without diabetes family history.

|  | Three generations of family history  (n=105) | No diabetes family history  (n=137) | *P* |
| --- | --- | --- | --- |
| Age at admission (years) | 30.51±6.51 | 33.99±5.21 | **0.000** |
| Age at diagnosis (years) | 25.39±6.31 | 30.80±5.87 | **0.000** |
| Duration of diabetes (years) | 4.00(1.00-8.25) | 1.00(0.30-5.00) | **0.000** |
| Male (%) | 58(55.24%) | 76(55.47%） | 0.971 |
| BMI (kg/m^2^) | 25.72±3.92 | 27.85±4.76 | **0.000** |
| WHR | 0.89±0.10 | 0.95±0.06 | **0.000** |
| HbA1c (%) | 8.80±2.15 | 9.70±2.20 | **0.002** |
| FPG (mmol/l) | 9.68±3.82 | 8.60±3.06 | **0.016** |
| SBP (mmHg) | 121.51±15.96 | 127.46±16.77 | **0.006** |
| DBP (mmHg) | 78.50±9.83 | 80.99±12.16 | 0.090 |
| C-peptide (ng/ml) | 2.08±0.83 | 2.13±1.09 | 0.701 |
| CR (umol/l) | 64.92±21.08 | 66.38±17.33 | 0.558 |
| BUN (mmol/l) | 4.82±1.72 | 4.44±1.50 | 0.069 |
| UA (mmol/l) | 350.56±83.39 | 359.85±90.05 | 0.487 |
| TBIL (umol/l) | 14.39±5.87 | 15.15±5.57 | 0.362 |
| DBIL (umol/l) | 2.24±1.16 | 2.78±1.68 | **0.004** |
| hsCRP (ng/ml) | 1.98(0.90-5.49) | 1.61(0.56-4.72) | 0.320 |
| TC (mmol/l) | 4.84±1.20 | 5.08±1.49 | 0.222 |
| TG (mmol/l) | 1.47(1.05-2.50) | 2.07(1.38-3.40) | **0.002** |
| LDL (mmol/l) | 2.98±0.97 | 2.99±1.10 | 0.976 |
| HDL (mmol/l) | 1.09±0.32 | 0.92±0.30 | **0.000** |
| HOMA-β | 52.81±37.58 | 65.36±52.96 | **0.043** |
| HOMA-IR | 1.95±1.02 | 1.88±1.08 | 0.612 |

BMI, body mass index; WHR, waist hip ratio; HbA1c, glycated hemoglobin; FPG, fasting plasma glucose; SBP, systolic blood pressure; DBP, diastolic blood pressure; CR, creatinine; BUN, blood urea nitrogen; UA, uric acid; TBIL, total bilirubin; DBIL, direct bilirubin; hsCRP, high sensitivity C-reactive protein; TC, total cholesterol; TG, triglycerides; LDL, low-density lipoproteins; HDL, high-density lipoproteins; HOMA-β: homeostatic model assessment indices for beta-cell function; HOMA-IR: homeostatic model assessment indices for insulin resistance.
